# Community Pharmacies Mood Intervention Study (CHEMIST): feasibility and external pilot randomised controlled trial protocol

**DOI:** 10.1186/s40814-019-0457-y

**Published:** 2019-05-29

**Authors:** Elizabeth Littlewood, Shehzad Ali, Jay Badenhorst, Della Bailey, Clare Bambra, Carolyn Chew-Graham, Elizabeth Coleman, Suzanne Crosland, Samantha Gascoyne, Simon Gilbody, Catherine Hewitt, Claire Jones, Ada Keding, Charlotte Kitchen, Dean McMillan, Caroline Pearson, Shelley Rhodes, Claire Sloan, Adam Todd, Michelle Watson, Cate Whittlesea, David Ekers

**Affiliations:** 1 Department of Health Sciences, University of York, York, YO10 5DD UK; 2 Whitworth Chemists Ltd, 2C Atkinson Way, Foxhill Industrial Estate, Scunthorpe, DN15 8QJ UK; 3 Institute of Health & Society, Newcastle University, Richardson Road, Newcastle Upon Tyne, NE2 4AX UK; 4 Research Institute, Primary Care and Health Sciences, Keele University, Staffordshire, ST5 5BG UK; 5 Public Health Team, Children & Adult Services, Durham County Council, County Hall, DH1 5UJ UK; 6 University of Exeter Medical School, University of Exeter, Exeter, EX1 2LU UK; 7 School of Pharmacy, King George VI Building, Queen Victoria Road, Newcastle Upon Tyne, NE1 7RU UK; 8 UCL School of Pharmacy, University College London, 29-39 Brunswick Square, London, WC1N 1AX UK; 9 Tees Esk and Wear Valleys NHS FT/University of York, Tarncroft House, Lanchester Road Hospital, Durham, DH1 5RD UK

## Abstract

**Feasibility study:**

Objectives:

- Refine a bespoke enhanced support intervention (ESI) (including self-help materials, intervention manual and training) for implementation by community pharmacy (CP) staff to people with sub-threshold depression and long-term conditions (LTCs) based upon evidence-supported interventions in primary care
- Develop and refine study procedures (recruitment strategies and set up, screening, participant recruitment, assessment, suitability of outcome measures and data collection procedures) for testing in the pilot study phase

Design: A case series/qualitative study

Setting: UK community pharmacy

Population: Adults with long-term health conditions who screen-positive for depression but who do not reach the threshold for DSM IV Moderate Depressive disorder

Intervention: Enhanced support intervention (ESI) delivered by an appropriately trained community pharmacy team member involving four to six sessions over four months. ESI is a modified form of an intervention within the collaborative care framework for sub-threshold depression validated in previous studies in UK primary care which appears suitable for implementation in community settings.

Sample size: 20–30 participants

Outcomes: Study implementation (recruitment and attrition rates), quality of data collection at baseline and 4 months and ESI adherence (number of contacts, DNA and drop out) as per objectives 1a/b

Qualitative evaluation: Semi-structured interviews with up to 10 participants and ESI facilitators and focus group(s) (range of pharmacy staff *n* = 8–10) will be conducted to explore the acceptability of the intervention and feasibility of the study, training and study procedures.

**External pilot study:**

Objectives:

- Quantify the flow of participants (eligibility, recruitment and follow-up rate)
- Evaluate proposed recruitment, assessment and outcome measure collection methods
- Examine the delivery of the enhanced support intervention in a community pharmacy setting (intervention uptake, retention and dose) to inform process evaluation
- Process evaluation, using semi-structured interviews with participants across a range of socio-economic settings, and pharmacy staff to explore the acceptability of the ESI within community pharmacy, elements of the intervention that were considered useful (or not) and appropriateness of study procedures

Design: Pilot randomised controlled trial, including a prospective economic and qualitative evaluation

Setting: As above

Population: As above

Intervention: As above with adaptations post feasibility study

Comparator: Usual care

Sample size: 100 participants

Outcomes: Data will be used to estimate recruitment, intervention delivery and study completion rates as per objectives 2a–d. Definitive estimates of the effectiveness of ESI will not be made.

Primary outcome: Depression severity (Patient Health Questionnaire 9) at four months.

Secondary outcomes: Patient acceptance, uptake and attrition. ICD10 depression status, anxiety (GAD 7), health-related quality of life (SF-12v2) and health-state utility (EQ5D 3L) will be measured at four months.

Economic evaluation: The incremental cost per QALY will be calculated from both the NHS and societal perspective.

Process evaluation: Using mixed methods, potential mediators/moderators of the intervention, the acceptability (to participants and pharmacy staff), barriers and facilitators to the use of ESI in community pharmacy, and impact on usual practice will be examined. Semi-structured interviews with approximately 30 study participants, 20 pharmacy staff and eight GPs near participating pharmacies will be conducted.

**Trial registration:**

ISRCTN: ISRCTN11290592

Protocol version number: Version 4.1 (dated 16th January 2018)

Study Sponsor Tees Esk and Wear Valleys NHS Foundation Trust

**Electronic supplementary material:**

The online version of this article (10.1186/s40814-019-0457-y) contains supplementary material, which is available to authorized users.

## Background

Depression accounts for 4.3% of the global disease burden and causes 63 million disability-adjusted life years annually. It is the largest cause of disease burden of all mental health problems and is set to become the highest amongst all health problems by 2030 [1]. Two to threefold increase in the prevalence of depression is found across the range of long-term conditions (LTCs) resulting in poorer outcomes, lower quality of life, a reduced ability to self-manage, substantial increased cost [2] and a significant contribution to health inequalities [3]. Sub-threshold depression, identified by a positive screen on the Whooley questions [4] and between two and four symptoms of depression, is highly prevalent and a major risk factor for progression to major depression [5]. Sub-threshold depression has comparable rates of associated excess mortality to major depression [6]. Estimates of the prevalence of sub-threshold depression in community samples range from 1.4 to 17.9% [7] and up to 20% in those people with LTCs. Clinical guidelines recommend brief psychological support [8]; however, psychological healthcare services struggle to meet demands of major depression and over 80% of ‘below threshold’ conditions remain untreated [9]. Psychological interventions reduce depressive symptoms in this population and reduce the incidence of major depression [10] but are not commonly available. Adopting a public health approach in at-risk groups such as those with LTCs may be an alternative to standard health services intervention; however, there is very limited evidence to support implementation.

Community pharmacies are ideally placed to offer opportunistic support to people with a range of health problems including low mood [11]. The Healthy Living Pharmacy programme is an example of extended roles of pharmacy teams, delivering for example smoking cessation and weight management interventions, through new innovative training programmes. These programmes lend support to the National Health Service (NHS) and Public Health England’s Five Year Forward View [12] calling for a radical upgrade in public health, including new partnerships that ‘break down barriers’ to support people with multiple health problems. The Marmot Review on Health Inequalities [13] recommended a focus on the mental health of people with long-term health problems due to the uneven socio-economic distribution and impact of such co-morbidities. The content of behavioural change/management approaches to sub-threshold depression shares much with other public health interventions such as smoking cessation or weight management (goal setting, facilitated self-help and diary keeping). Therefore, these interventions targeted at sub-threshold depression may also be suited to delivery by those staff delivering other public health behavioural change programmes. Given that 90% of people live within 20 min walk of their local pharmacy, especially within areas of high social deprivation [14] and with high rates of ‘footfall’ of people with LTCs, community pharmacies have a unique position to offer enhanced support for any co-morbid sub-threshold depression alongside other health promotion activities. High-quality evidence is needed to inform the design and delivery of such services. The Community Pharmacies Mood Intervention Study (CHEMIST) seeks to adapt ‘what works’ for people with sub-threshold depression in primary care and examine if this can be translated to the important public health setting of community pharmacy. This feasibility and pilot randomised controlled trial (RCT) study will address the potential for a behavioural change intervention and associated study procedures to work in this innovative public mental health setting in preparation for a definitive RCT. If this public health intervention is shown to have potential, a definitive RCT would also need to examine the depression prevention value of the intervention, which is of particular benefit to the individual, healthcare system and society [15].

Reviews of the literature indicate existing evidence is not sufficiently robust, or experience sufficiently developed, to support a definitive trial at this stage in the evolution of this public health intervention. CHEMIST therefore has two sets of objectives. The first set of objectives to be met in the feasibility phase is to refine our intervention (enhanced support intervention [ESI]) and research procedures. The second set of objectives to be met in the pilot RCT phase is to estimate study recruitment, acceptability and retention.

### Feasibility study objectives

- Refine the bespoke enhanced support intervention (ESI) (including self-help materials, intervention manual and training) for implementation by community pharmacy staff for use with people with sub-threshold depression and LTCs based upon evidence-supported interventions in primary care.
- Develop and refine study procedures (community pharmacy set-up and recruitment strategies; participant screening, recruitment and assessment; suitability of outcome measures; and data collection procedures) for testing in the pilot RCT phase.

### External pilot RCT objectives

- Quantify the flow of participants (eligibility, recruitment and follow-up rate).
- Evaluate proposed recruitment, assessment and outcome measure collection methods.
- Examine the delivery of the ESI in a community pharmacy setting (intervention uptake, retention and dose) to inform the process evaluation.
- Process evaluation, using semi-structured interviews with participants, pharmacy staff and general practitioners (GPs) across a range of socio-economic settings to explore the acceptability of the ESI within the community pharmacy setting, elements of the intervention that were considered useful (or not), and appropriateness of study procedures.

## Methods—feasibility study

### Design

A case series study with nested qualitative evaluation.

### Setting

Community pharmacies in the North of England

### Study population

Inclusion criteria: Adults (18+) with sub-threshold depression (screen positive with two to four depression symptoms confirmed by a diagnostic assessment tool) and one or more LTCs.

Exclusion criteria: People who have alcohol or drug dependence, or cognitive impairment, or have bipolar disorder/psychosis/psychotic symptoms, or are acutely suicidal, or those currently in receipt of psychological therapy.

### Recruitment

Recruitment to sub-threshold depression studies within community pharmacy is untested in the UK. Therefore, a key objective of the feasibility study will be to refine potential recruitment approaches to test in the external pilot RCT. Three approaches will be used.

1. Pharmacy recruitment. Potential participants will be identified via medication use linked to LTC and/or other pharmacy use. They will be provided with a study information pack either directly, attached to home delivery prescriptions or via pharmacy mail outs.
2. Publicity recruitment. Posters will be placed in community pharmacies, general practices and local community settings advertising the study with a central contact point for the study team. Interested people will be invited to contact the study team either by phone, email or via the study website.
3. Primary care recruitment. General practice (GP) database searches will be used to identify potential participants (those with one or more LTC) and the study information pack will be mailed out by the practices.

The study information pack will include an invitation letter, a participant information sheet, a consent form, a background information sheet and a stamped addressed envelope for return to the study team.

These procedures will be refined in the feasibility phase to facilitate use and evaluation in the pilot RCT.

### Diagnostic confirmation

The Mini International Neuropsychiatric Interview (MINI) [16] will be used to establish the presence or absence of depression symptoms and disorder (sub-threshold/major depression). Diagnostic interviews will be carried out by a trained researcher over the telephone.

### Intervention

Enhanced support intervention (ESI): designed specifically for those with sub-threshold depression and LTCs to be delivered over four to six sessions in a 4-month period. Sessions will be delivered via telephone or face to face in the privacy of pharmacy consulting rooms. The ESI will be adapted from training/treatment materials used in previous sub-threshold depression research [17]. The ESI consists of four main elements.

1. Behavioural activation (BA) focussed self-help support. A simple psychological approach focussed upon identifying life changes that have a detrimental impact on psychologically healthy activities and scheduling to stay well. This will be facilitated by a printed self-help workbook and supported by a trained pharmacy staff member (ESI facilitator).
2. Proactive follow-up. Participants will be provided with a BA self-help workbook and an ESI facilitator would telephone/meet the participant at regular intervals to support its use.
3. Symptom monitoring. The ESI facilitator will monitor symptoms at each support session using the depression scale from the Depression Anxiety Stress Scale (DASS) which is widely used/validated in a UK community context [18]. It is brief, simple to score, with clear clinical cut off scores (non/mild/moderate/severe).
4. Decision supported signposting. Scores on the DASS will be used to guide decision-making by the ESI facilitator, guided by supervision delivered by clinical members of the study team. Where risk or significant clinical deterioration is noted, the participant would be supported to access more formal healthcare interventions.

The ESI will be delivered by pharmacy support staff (ESI facilitators) experienced in the delivery of extended roles (such as smoking cessation using behavioural change approaches) and/or training to Royal Society of Public Health standard (Understanding Health Improvement Level 2). Fidelity will be supported by ESI facilitator manuals and comprehensive face-to-face training adapted from those used in previous studies. In addition, a bespoke competency assessment will be used (based upon experience from previous studies) at the end of the intervention training to ensure ESI facilitators are able to support participants in the intervention. With participant consent, audio recordings of the intervention support sessions will be undertaken. A random selection of recordings (10–20%) across the different phases of the intervention (early/late) will be collected from each ESI facilitator and independently reviewed.

### Outcome measures

Depression severity will be measured by the Patient Health Questionnaire 9 (PHQ9) [19]. Widely used in clinical trials and settings, the PHQ9 provides excellent internal and external validity and specificity/sensitivity in a UK population [20]. The PHQ9 would be the primary outcome measure in a definitive RCT.

Secondary outcome measures will include prevention of depression (PHQ9 less than 10), anxiety (GAD7) [21], somatic symptoms (PHQ15) [22], quality of life (SF-12v2) [23], health-state utility (EQ5D-3 L) [24] and healthcare utilisation via an adapted version of the Adult Service Use Schedule (AD-SUS).

#### Process measures

Moderators: depression severity (PHQ9), age of onset (MINI), number of episodes (MINI), LTC and socioeconomic status will be collected at baseline interview. Mediators: number of contacts, level of activation using the Behavioural Activation for Depression Scale (BADS) collected at treatment sessions [25].

Baseline demographic measures: DOB, ethnicity, education level, socioeconomic status and sex.

### Assessment and follow-up

Data will be collected at screening/eligibility, baseline and four  months post-baseline. Due to the nature of the feasibility study, blinding to allocation is not possible.

### Sample size

No formal sample size calculation has been undertaken for the feasibility study. A sample size of 20–30 participants recruited will be sufficient to meet feasibility phase objectives.

### Outcomes and data analysis

As this is the feasibility phase, study implementation (recruitment and attrition rates, quality of data collection) will be assessed at baseline and four months, along with ESI adherence (number of contacts, DNA and drop out), as per the feasibility objectives. Rates will be reported descriptively.

### Qualitative evaluation

In-depth interviews will be conducted with up to ten patient participants (purposively sampled across pharmacy location and with a mix of LTCs, from different areas of deprivation) to explore the acceptability of the content and location of the ESI. Interviews with approximately ten ESI facilitators will be conducted following training and again at the end of the feasibility study. A focus group will be conducted with pharmacy staff to explore the feasibility and acceptability of the training, intervention and study procedures.

Existing literature will be used to develop topic guides. The Normalisation Process Theory (NPT) approach [26] will be used to sensitise topic guides and provide a framework for analysis and interpretation of data in order to identify the barriers and facilitators to implementation. This approach has been used in previous studies [27].

The analysis will inform adaptations to the intervention materials, training and study procedures for the pilot RCT.

### Qualitative evaluation recruitment

Recruitment of patient participants for qualitative interviews will be included in the information and consent process for the feasibility study.

Staff involved in CHEMIST particularly ESI facilitators will be contacted directly by the study team by letter and provided with the staff participant information sheet and consent form. They will be provided with pre-paid envelopes to return the documentation directly to the study team. This process will be carried out independently of the pharmacy to ensure it is free from coercion from employers or co-workers. All data collected will be anonymised by the qualitative research team prior to reporting to ensure the confidentiality of participants.

## Methods—external pilot study

### Design

An external pilot RCT including nested process evaluation and economic evaluation.

### Setting

Community pharmacies in the North of England sampled across quintiles of area-level deprivation.

### Study population

As described in the feasibility study above.

### Recruitment

Recruitment methods will include those described in the feasibility study above, but with refinements following learning from feasibility phase.

### Outcome measures

As described in the feasibility study above but with the exclusion of the PHQ15.

### Assessment and follow-up

Participants will be offered the option of completing baseline and follow-up questionnaires over the telephone or via post. Due to the nature of the study, participants and those delivering the intervention will be unblinded to allocation. Researchers carrying out follow-up assessments will be blinded to treatment allocation.

### Intervention

As described in the feasibility study above but with adaptations made following the feasibility study.

### Comparator

The control group will be the usual primary care management of sub-threshold depression and other local community provision.

### Sample size

Sample size calculations are based on estimating attrition and the standard deviation of the primary outcome. Assuming 20% of participants are lost to follow-up with a sample size of 100, then the 95% confidence interval for this level of attrition will be the observed difference ± 8 percentage points (i.e. between 12 and 28%) (%) [28]. Hence, an external pilot trial of 100 participants should ensure robust estimates of recruitment and follow-up in this population. Furthermore, an external pilot study of 80 measured subjects will provide robust estimates of the standard deviation of the outcome measure in this population to inform the sample size calculation for a subsequent larger definitive fully powered trial [29].

### Randomisation and allocation concealment

Individual independent randomisation will be used. Randomisation will be carried out by the York Trials Unit (YTU), University of York, online randomisation service (URL:www.yorkrand.com/), independently of the trial team. One hundred participants will be randomised on a 1:1 basis to either the intervention group (50) or control group (50) following completion of the baseline interview.

### Outcome and data storage and analysis

Outcome data will be stored securely at the Department of Health Sciences, University of York, following strict confidentiality and data storage standard operating procedures (SOPs) and protocols. Pilot RCT data will be scanned and validated at the York Trials Unit as defined by YTU SOPs and protocols. Data will be stored on the YTU system and will be the responsibility of the University of York and sponsor (TEWV NHS FT) as set out in a collaboration agreement. Requests for access to data will be considered in line with YTU and sponsor SOPs.

The flow of participants through the trial will be detailed in a CONSORT flow diagram (see Additional file 1). The number of people screened, randomly assigned, receiving the intervention, completing the study protocol and providing outcome data will be summarised overall and by trial arm. The number of individuals withdrawing from the intervention and/or the trial and any reasons for withdrawal will be summarised by trial arm. To quantify the acceptability of the intervention, the number of sessions attended will also be summarised. The primary outcome of this study will be reported descriptively by treatment arm using mean, standard deviation (SD), 95% confidence intervals, median and 25th and 75th percentiles.

While the main aim of this study is to establish practicality, feasibility, recruitment rates and key parameters for the sample size in order to inform a full-scale trial, and although it is unlikely that the small sample size will result in effectiveness being established, we will none the less test the primary outcome to mimic practice for full-scale trial. Results from this analysis will be treated as unreliable and interpreted with caution [30, 31].

The two groups will be compared for the primary outcome (PHQ-9 at 4 months follow-up) using linear regression with adjustment for important baseline covariates. Estimates from the model and 95% CI will be presented. We may also explore the impact of pharmacy effects. The potential impact of ‘pharmacy effects’ will be quantified using intraclass correlation coefficient (ICC) estimates with 95% confidence intervals and the average caseload per pharmacy ESI facilitator. All secondary outcomes will be summarised descriptively by treatment arm using mean, SD, 95% confidence intervals, median, 25th and 75th percentiles for continuous outcomes, and the number of events and percentages for categorical data.

### Health economics

The economic analysis will evaluate the feasibility of collecting resource use and health-related quality of life (HRQoL) data in people with long-term health conditions and sub-threshold depression. It will enable us to identify relevant resource use categories for the cost-effectiveness analysis and evaluate the feasibility and challenges of measuring costs and outcomes in the study population. The feasibility of collecting intervention costs and acquiring unit cost data for all resource use categories will be assessed. The primary analysis will focus on questionnaire completion rates and item-level rates of missing data at each time point for both resource use questionnaire and quality of life instruments. Also, the level of resource use and utility levels (and quality-adjusted life years) will be presented for each group. The feasibility of conducting a cost-effectiveness analysis using resource use and quality of life instruments data will be evaluated. A cost-utility analysis using the EQ-5D-3L and SF-12v2 responses which will be converted into utility scores using UK population algorithms [32] will be conducted. Then, the area under the curve approach will be used to calculate quality-adjusted life years (QALYs) for both groups. To estimate the incremental difference in costs and QALYs, a regression approach will be used which will control for baseline characteristics of patients, particularly baseline utility. The regression coefficient will provide the estimate of the incremental difference in QALYs and costs between the treatment and control arms. A bootstrap approach will be used to get the confidence interval around the incremental cost-effectiveness ratio. The results will be presented on the cost-effectiveness plane and as cost-effectiveness acceptability curve to evaluate the probability of being cost-effective against the commonly used willingness-to-pay threshold in the UK.

### Process evaluation

As per UK MRC recommendations, a mixed methods approach to process evaluation [33] will be used to consider fidelity, implementation, mechanisms and context. Descriptive statistics will be used to describe elements of the intervention (participants, dose and intervention content). The moderational analysis will examine baseline variables that may moderate outcomes such as age, depression onset age, number of episodes and socioeconomic status. The mediational analysis will investigate hypothesised mechanisms of change such as number of sessions, content of scheduled contacts (from pharmacy ESI facilitator records) and changes in activation (BADS) using established methodology [34]. As this is an external pilot RCT, it is considerably underpowered to undertake these evaluations so data will be summarised and used to refine process evaluation design to inform a future large-scale definitive RCT.

A process evaluation using qualitative methods will be used to explore acceptability and important elements of the intervention from the perspective of the participants. A purposive sample of approximately 15 participants who completed the intervention will be invited to participate in a semi-structured interview (recruitment and data collection will continue until category saturation has been achieved). Sampling will ensure that participants with a range of age and gender are sampled and from a range of socio-economic groups. The interviews will explore the acceptability of case-finding for sub-threshold depression, receiving the enhanced support intervention (ESI) within a community pharmacy setting, impact on relationship with general practice and research procedures. A sample of participants who dropped out (had less than two sessions) will also be invited to participate in a semi-structured interview to explore reasons for drop out. It is likely approximately 15 interviews will be needed to achieve data saturation [35].

Sampling will be ensured in areas of higher deprivation (i.e. bottom two quintiles of index of multiple deprivation (IMD)), to enable us to explore perspectives of people particularly from lower socioeconomic status (SES). Perceptions of the appropriateness of the intervention for people with poor health literacy and from lower socioeconomic status (SES) will be explored. All interviewees will be interviewed at a time and place convenient to them (e.g. at home, in the pharmacy, GP surgery or by telephone).

Interviews with patient participants will be conducted after the primary outcome data has been collected.

Interviews with approximately 20 pharmacy staff (across a broad range of pharmacy roles) will examine acceptability and important elements of the intervention, training and supervision, barriers/facilitators to participation and implementation, and impact on pharmacy practice.

Interviews with approximately eight local GPs will explore knowledge and perspectives of the intervention, impact on routine practice and how this intervention might be implemented in practice.

Semi-structured interviews offer opportunities to cover in-depth, a range of topics relevant to the research questions, whilst also allowing for exploration and probing of additional issues raised during the interviews. Interviews topic guides will vary by group; these may be amended iteratively as interviews progress but will contain core questions highlighted in the feasibility study. Interviews will last up to an hour and will be digitally recorded with consent and transcribed verbatim, the transcripts forming the data for analysis. Coding will be undertaken independently by members of the qualitative research team with meetings to ensure emerging codes remain grounded in the original data. Initial analysis will use the principles of constant comparison, which will allow modification of the topic guides and analysis across the data sets [34]. Further analysis will use the principles of framework analysis and NPT [26].

### Process evaluation—recruitment

Recruitment of patient participants will be as described in the feasibility study above.

ESI facilitators will be provided with the staff participant information sheet and consent form upon completion of their intervention training. Remaining pharmacy staff will be contacted by letter and provided with the same study information. A reminder letter will be sent once the intervention delivery has commenced in each participating pharmacy. All pharmacy staff will be provided with pre-paid envelopes to return the consent form directly to the study team. Processes will ensure participation is confidential and free from any coercion by employers or co-workers.

The GPs of recruited participants and GPs in participant identification sites will be contacted by letter and invited to take part. They will be provided with a participant information sheet, consent form and pre-paid envelope to return to the study team.

### CHEMIST study governance

The study will be led by the Chief Investigator (DE) with day-to-day management provided by the Trial Manager (EL). There will be an independent Trial Steering Group (TSG) and Data Monitoring and Ethics Committee (DMEC) as per NIHR recommended constitution. The sponsor will provide oversight and audit of the study as per local standard operating procedures. Serious adverse events will be collected and monitored as per local standard operating procedures.

Protocol amendments will be managed via the Health Research Authority, Research Ethics Committee and sponsor approvals process during the study.

### Dissemination

The study will be disseminated via peer-reviewed journals and conference presentations. Plain English summaries will be produced and distributed via partner stakeholders and to study participants. Results will be used to assess if and how a fully powered RCT is practical.

## Discussion

Community pharmacies are increasingly used to deliver health promotion activities and have regular contact with people with long-term health conditions. The use of community pharmacy in research, and in particular in depression research, is new and as such requires testing prior to conducting large scale randomised controlled trial. The CHEMIST feasibility and pilot RCT study is an innovative design in this novel area. The methods outlined will produce data on how and if future trials might be conducted and will be disseminated through conference presentation and peer-reviewed open access publication.

### CHEMIST study status

At time of writing the CHEMIST study is still in its recruitment phase and process evaluation has commenced.

## Additional file

Additional file 1: CHEMIST pilot RCT consort flow chart. (DOCX 40 kb)

## Abbreviations

BA: Behavioural activation
CI: Confidence interval
CP: Community pharmacy
DMEC: Data Monitoring and Ethics Committee
DNA: Did not attend
DOB: Date of birth
ESI: Enhanced support intervention
GP: General practice
GPs: General practitioner
ICC: Intraclass correlation coefficient
IMD: Index of multiple deprivation
LTC: Long-term conditions
MINI: Mini International Neuropsychiatric Interview
NHS: National Health Service
NIHR: National Institute of Health Research
NPT: Normalisation Process Theory
PHR: Public Health Research
QALY: Quality-adjusted life year
RCT: Randomised controlled trial
SD: Standard deviation
SES: Socioeconomic status
SOP: Standard operating procedure
TSG: Trial Steering Group
YTU: York Trials Unit

## References

[1] World Health Organisation (2008). The global burden of disease: 2004 update.

[2] Naylor C, Parsonage M, McDaid D, Knapp M, Fossey M, Galea A. Long term conditions and mental health: the cost of co-morbidities. London: The Kings Fund; 2012.

[3] Mercer SW, Watt GCM (2007). The inverse care law: clinical primary care encounters in deprived and affluent areas of Scotland. Ann Fam Med.

[4] National Institute for Health and Clinical Excellence (2011). Common mental health disorders: identification and pathways to care.

[5] Cuijpers P, de Graaf R, van Dorsselaer S (2004). Minor depression: risk profiles, functional disability, health care use and risk of developing major depression. J Affect Disord.

[6] Cuijpers P, Vogelzangs N, Twisk J, Kleiboer A, Li J, Penninx BW (2013). Differential mortality rates in major and subthreshold depression: meta-analysis of studies that measured both. Br J Psychiatry.

[7] Rodriguez M, Nuevo R, Chatterji S, Ayuso-Mateos J (2012). Definitions and factors associated with subthreshold depressive conditions: a systematic review. BMC Psychiatry.

[8] National Institute for Health and Clinical Excellence (2009). NICE clinical guideline 91: depression in adults with a chronic physical health problem.

[9] McManus S, Meltzer H, Brugha T, Bebbington P, Jenkins R. Adult psychiatric morbidity in England 2007: results of a household survey. Leeds: NHS Digital; 2009.

[10] Cuijpers P, Koole SL, van Dijke A, Roca M, Li J, Reynolds CF (2014). Psychotherapy for subclinical depression: meta-analysis. Br J Psychiatry.

[11] NHS confederation (2013). Health on the high street: rethinking the role of community pharmacy.

[12] NHS and Public Health England’s Five Year Forward View Plan. https://www.england.nhs.uk/publication/nhs-five-year-forward-view/. Accessed 2 Sept 2016.

[13] Marmott M (2010). The Marmot review final report: fair society, healthy lives.

[14] Todd A., Copeland A., Husband A., Kasim A., Bambra C. (2014). The positive pharmacy care law: an area-level analysis of the relationship between community pharmacy distribution, urbanity and social deprivation in England. BMJ Open.

[15] van Zoonen K, Buntrock C, Ebert D, Smit F, Reynolds C, Beekman A (2014). Preventing the onset of major depressive disorder: a meta-analytic review of psychological interventions. Int J Epidemiol.

[16] Sheehan DV, Lecrubier Y, Sheehan KH, Amorim P, Janavs J, Weiller E (1998). The Mini-International Neuropsychiatric Interview (M.I.N.I.): the development and validation of a structured diagnostic psychiatric interview for DSM-IV and ICD-10. J Clin Psychiatry.

[17] Gilbody S, Lewis H, Adamson J, Atherton K, Bailey D, Birstwistle J (2017). Effect of collaborative care vs. usual care on depressive symptoms in older adults with subthreshold depression: the CASPER randomized clinical trial. JAMA.

[18] Henry JD, Crawford JR (2005). The short-form version of the Depression Anxiety Stress Scales (DASS-21): construct validity and normative data in a large non-clinical sample. Br J Clin Psychol.

[19] Kroenke K, Spitzer R, Williams J (2001). PHQ9: Validity of a brief depression measure. J Gen Intern Med.

[20] Gilbody S, Richards D, Barkham M (2007). Diagnosing depression in primary care using self-completed instruments: UK validation of PHQ–9 and CORE–OM. Br J Gen Pract.

[21] Spitzer R, Kroenke K, Williams J, Lowe B (2006). A brief measure for assessing generalized anxiety disorder: the GAD-7. Arch Intern Med.

[22] Kroenke K, Spitzer RL, Williams JBW (2002). The PHQ-15: validity of a new measure for evaluating the severity of somatic symptoms. Psychosom Med.

[23] Ware J, Kosinki M, Keller SA (1996). 12-item short-form health survey: construction of scales and preliminary tests of reliability and validity. Med Care.

[24] EuroQol Group (1990). EuroQol: a new instrument for the measurement of health-related quality of life. Health Policy.

[25] Manos RC, Kanter JW, Luo W (2011). The behavioral activation for depression scale–short form: development and validation. Behav Ther.

[26] May C, Finch T (2009). Implementing, embedding, and integrating practices: an outline of normalization process theory. Sociology..

[27] Coupe N, Anderson E, Gask L, Sykes P, Richards D, Chew-Graham C (2014). Facilitating professional liaison in collaborative care for depression in UK primary care; a qualitative study utilising normalisation process theory. BMC Fam Pract.

[28] Hertzog MA (2008). Considerations in determining sample size for pilot studies. Res Nurs Health.

[29] Teare MD, Dimairo M, Shephard N, Hayman A, Whitehead A, Walters SJ (2014). Sample size requirements to estimate key design parameters from external pilot randomised controlled trials: a simulation study. Trials..

[30] Lancaster GA, Dodd S, Williamson PR (2004). Design and analysis of pilot studies: recommendations for good practice. J Eval Clin Pract.

[31] Arain M, Campbell MJ, Cooper CL, Lancaster GA (2010). What is a pilot or feasibility study? A review of current practice and editorial policy. BMC Med Res Methodol.

[32] Dolan P (1997). Modeling valuations for EuroQol health states. Med Care.

[33] Moore GF, Audrey S, Barker M, Bond L, Bonell C, Hardeman W (2015). Process evaluation of complex interventions. Med Res Council Guidance.

[34] Kraemer H, Wilson G, Fairburn CG, Agras W (2002). Mediators and moderators of treatment effects in randomized clinical trials. Arch Gen Psychiatry.

[35] Silverman D (1997). Qualitative research: theory, method and practice.

