# Supplementary figures and images for: Community Pharmacies Mood Intervention Study (CHEMIST): feasibility and external pilot randomised controlled trial protocol
Source: Pilot Feasibility Stud. 2019 May 29;5:71. doi: 10.1186/s40814-019-0457-y (PMC6540405; doi:10.1186/s40814-019-0457-y)

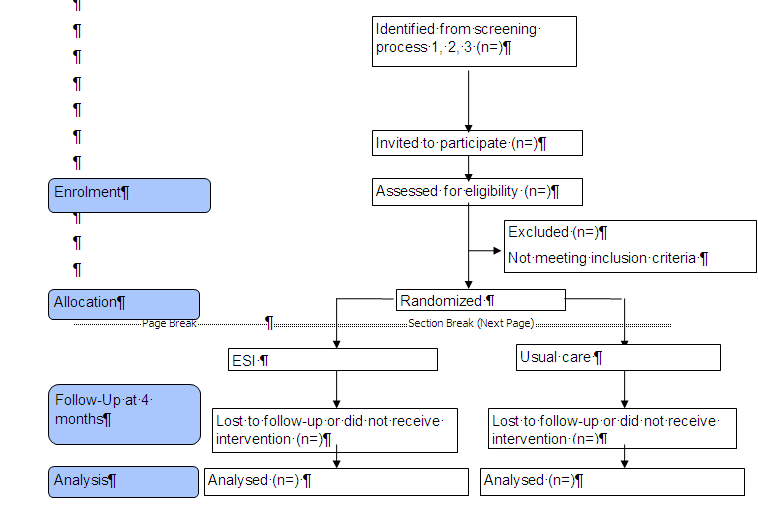

Supplement: Supplementary file 1 — CHEMIST pilot RCT consort flow chart. (DOCX 40 kb) [file 40814_2019_457_MOESM1_ESM.docx]
